# Implications of Stimulation Modality and Control Condition on BOLD Response: An Example From the MOUS Dataset

**DOI:** 10.1162/NOL.a.25

**Published:** 2025-12-01

**Authors:** Anna-Lisa Schuler, Ella Teuscher, Nicola Filippini

**Affiliations:** Max Planck Institute for Human Cognitive and Brain Sciences, Leipzig, Germany; Medical University of Vienna, Vienna, Austria; IRCCS San Camillo Hospital, Venice, Italy

**Keywords:** auditory, control condition, fMRI, modality, sentence processing, visual

## Abstract

The choice of control conditions can significantly influence the outcomes of functional MRI (fMRI) studies. Moreover, especially in language experiments, the sensory modality (auditory, visual) of stimuli might have an influence on experimental results. In this study we leverage a repository dataset (Mother of Unification Studies; MOUS), to systematically investigate the influence of control condition and stimulation modality on fMRI results during sentence processing. Here, we explored fMRI data of 187 subjects that underwent sentence comprehension with either auditory or visual task presentation (reading or listening). Sentences were either complex, including a relative clause, or simple, not including a relative clause. Control conditions were randomly scrambled words as constructed out of the latter sentence conditions. While auditory stimulation resulted in strong activation changes in the bilateral auditory cortices, visual stimulation revealed stronger activation changes in the anterior temporal lobe if compared to simple and complex words, but not simple sentences. A direct comparison between the auditory and visual modality revealed stronger involvement of the primary auditory cortices for auditory stimulation and left inferior frontal gyrus for visual stimulation over all four conditions (complex sentences, simple sentences, complex words, simple words). The results of this study suggest that stimulation modality and control condition strongly influence sentence processing fMRI results. Future fMRI studies should bear this in mind for study planning.

## INTRODUCTION

The selection of stimulus material is at the core of task-based functional magnetic resonance imaging (fMRI) studies. This includes stimulation modality (auditory, visual) and selection of a control condition. Whereas stimulation modality is especially an issue in the context of linguistic studies, the selection of a control stimulus is of universal concern for task-based fMRI studies.

With respect to stimulation modality, a recent comprehensive meta-analysis over all fMRI studies addressing the linguistic domain (Turker et al., 2023) revealed a strong bilateral superior temporal cortex (STG) involvement for auditory as opposed to visual stimulation, suggesting predominant primary auditory processing. The opposite contrast revealed stronger involvement of the frontal eye field, superior parietal lobule, primary visual cortex, posterior middle temporal gyrus and ventral part of posterior temporal gyrus, indicating predominant involvement of visual attention and symbol processing.

In terms of syntactic processing, the role of the inferior frontal gyrus (IFG) has furthermore been debated, with some studies suggesting modality specific activation changes in this structure for syntactic processing (Rogalsky et al., 2015; Rogalsky & Hickok, 2011).

While stimulation modality poses a first source of variance, the proper selection of a control task is a matter of paramount importance in the face of interpretability of the contrasts of interest. The simplest way to design a task is to contrast test items with an implicit baseline, that is, against brain activity with no stimulus applied. This results, however, in a prominent activation change in primary visual or auditory cortices depending on stimulation modality. Choosing a control condition might then take into account the increasing complexity of the task in question. Exemplary for syntactic processing, these might be a linguistically meaningless stimulus (e.g., visual or auditory noise; Hwang et al., 2006; Pellegrino, Pinardi, et al., 2022; Pellegrino, Schuler, et al., 2022; Schuler et al., 2023), a task that accounts for phonemic processing (e.g., scrambled auditory strings or letters; Benjamin et al., 2017; Berl et al., 2006; Vasileiadi et al., 2023), or a task that accounts for semantic processing (increasing the complexity of a syntactic structure). Whereas these are theoretical considerations, in application there is no perfect control task that allows a clean subtraction of only primary sensory, phonetic, semantic, or syntactic features, always warranting caution in interpretability of task-based fMRI contrasts.

In this study, we are leveraging large-scale repository data investigating syntax processing employing different stimulation modalities (auditory and visual) on the same type of stimuli while employing four different task conditions (Schoffelen et al., 2019). The Mother of Unification Studies (MOUS) dataset features a total of 204 participants that underwent magnetoencephalography (MEG) and fMRI during sentence processing either via visual or auditory processing. It features four different task conditions: understanding complex sentences (featuring a relative clause), understanding simple sentences (not featuring a relative clause), and scrambled sentences of the two syntactic conditions on a word level (complex words, simple words). It therefore provides an ideal use case for the influence of stimulus modality and influence of control condition. The aim of this study is to demonstrate how applying different control conditions in fMRI will affect study outcomes. Moreover, using this dataset, we are able to show how control condition interacts with stimulation modality.

## MATERIALS AND METHODS

### Study Participants

All participants included in this study were taken from the publicly available MOUS dataset (Schoffelen et al., 2019). These data were collected at the Donders Centre for Cognitive Neuroimaging and are publicly available on the Donders Institute Repository (https://data.donders.ru.nl). The study was approved by the local ethics committee (CMO; Committee on Research Involving Human Subjects in the Arnhem-Nijmegen region–CMO2014/288) and followed guidelines of the Helsinki declaration. All participants provided written informed consent prior to data acquisition for the study.

All participants with recorded information for age and sex, availability of structural MRI, task-based and resting fMRI data, and appropriate log files (acquired during the task fMRI and necessary to recreate the variables of interest to model the blood oxygen-level dependent [BOLD] signal during stimulus presentation) were included in the analyses. All participants were native Dutch speakers.

### fMRI Experimental Design

**Figure 1.**
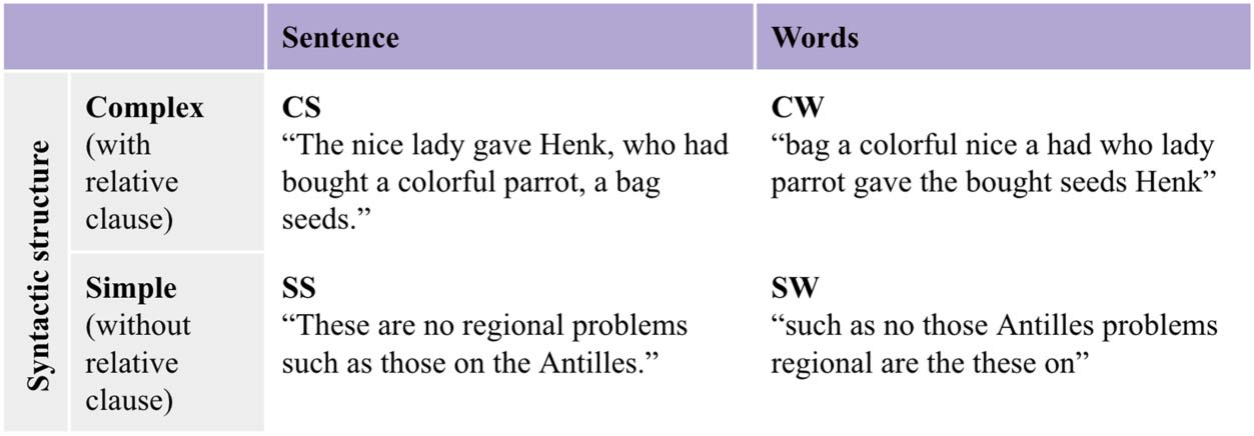
Experimental conditions. Examples correspond to English translations of the original Dutch stimuli.

The MOUS dataset was specifically designed to investigate brain areas involved in language processing. Within an experimental fMRI session, 60 stimuli were presented divided into 12 blocks. Each block consisted of normal sentences or scrambled words derived from the same sentences. Moreover, the sentences were either complex, featuring a relative clause to increase syntactic difficulty, or simple, containing only a main clause to obtain a simpler syntactic structure. Relative clauses were mostly subject-first with a few sentences that were object-first. Four main explanatory variables can be identified and used to model the BOLD signal: complex sentence (CS), simple sentence (SS), complex words (CW), and simple words (SW; Figure 1).

The word condition was created by scrambling the words from the sentences such that three or more consecutive words did not form a coherent fragment. All sentences varied between 9 and 15 words in length. The mean sentence length for complex sentences was 11.5 words and the mean for simple sentences 11.48 words, guaranteeing balanced stimulus material. Across participants, all stimuli were presented the same number of times in the sentence and in the scrambled list condition. The starting block type (either normal sentence or scrambled words) was randomized across participants. To check for compliance, 20% of the trials were followed by a yes/no question about the content of the previous normal sentence/scrambled word list. Half of the questions on the sentences addressed the content of the sentence (e.g., *Did grandma give a cookie to the girl?*), whereas the other half, and all of the questions about the scrambled word lists, addressed one of the main content words (e.g., *Was the word ‘grandma’ mentioned?*). Subjects answered the question by pressing a button for yes/no with their left index and middle finger, respectively. At the start of each block there was a 1,500 ms presentation of the block type: normal sentence or scrambled words. The intertrial interval was jittered between 3,200 and 4,200 ms. During this period, an empty screen was presented, followed by a fixation cross. The visual presentation rate of the stimuli was determined in relation to the duration of the audio recording of spoken versions of the sentences and the word lists (audiodur), taking into account both the number of letters (sumnletters) and words (nwords) in the whole sentence and the number of letters within each word (nletters). The duration of a single word (in ms) was determined as (nletters/sumnletters) ⁕ (audiodur + 2,000–150 ⁕ nwords).

Sentences contained target nouns that were either at the third or thirteenth position. They were matched based on frequency (SUBTLEX-NL; Keuleers et al., 2010) and word length. Auditory stimuli were recorded by a Dutch native speaker with neutral prosody. For further reading on stimulus material please refer to Schoffelen et al. (2019).

Prior to the task, subjects read a written instruction of the task and were allowed to ask questions for clarification. To familiarize the subjects with the task, participants had a practice task run with different stimuli from the actual study task.

**Figure 2.**
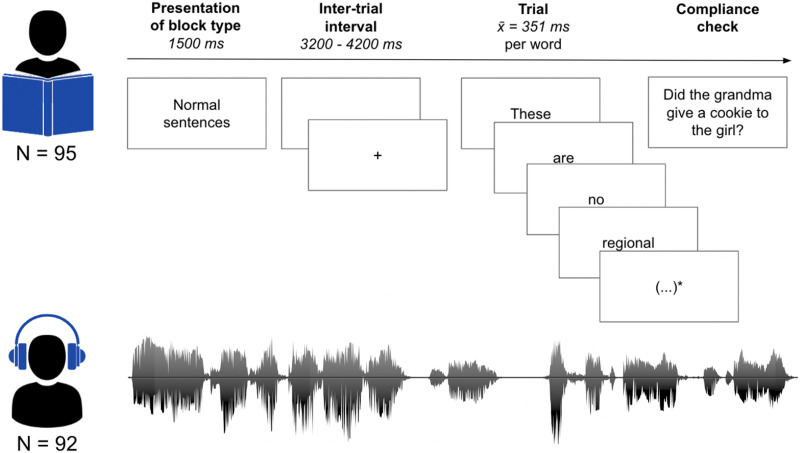
Example of a trial in the visual and auditory modality. In the visual modality, sentences or word lists were presented word by word with a minimum of 300 ms and maximum of 1,400 ms, depending on word length. Words were separated by an empty screen of 300 ms. Trials were separated by an intertrial interval of 3,200–4,200 ms; 20% of the trials were followed by compliance checks, regarding the content or a word of the trial. Participants replied by pressing a button for “yes” or “no” with their left index and middle finger, respectively. Before each block, the stimulus type was announced. This example corresponds to an English translation of an original trial in the Dutch language. Asterisk indicates the stimulus continues here.

Participants in the auditory condition received stimuli via plastic tubes and earpieces to both ears, whereas the participants in the visual group received stimuli displayed on an LCD projector. Programming of the paradigms was carried out using Presentation software (Version 16; Neurobehavioral Systems, 2025). An overview of the experimental course is given in Figure 2.

### MRI Data Acquisition

Scanning was performed using a 3-T Siemens Trio scanner with a 32-channel head coil. Structural and functional sequences were as follows.

**Task-based fMRI**. Language task was assessed from a single run using a T2*-weighted echo planar imaging (EPI) sequence with partial brain coverage (TR = 2,000 ms, TE = 35 ms, flip-angle = 90°, voxel dimensions = 3.5 × 3.5 × 3 mm^3^).
**Resting fMRI**. Whole-brain functional imaging was performed using a T2*-weighted EPI sequence with whole-brain coverage (TR = 1,680 ms, TE = 30 ms, flip-angle = 70°, voxel dimensions = 3.5 × 3.5 × 3 mm^3^). Subjects were instructed to lie in dimmed light with their eyes open, think of nothing in particular, and not to fall asleep during the data acquisition.
**Structural MRI**. High-resolution 3D T1-weighted MRI scans were acquired using a magnetization-prepared rapid gradient echo (MPRAGE) sequence (TR = 2,300 ms, TE = 3.03 ms, flip-angle = 8°, voxel dimensions = 1 mm^3^ isotropic).

Further details about the MRI protocol can be found in Schoffelen et al. (2019).

### Imaging Data Preprocessing and Statistical Analysis

MRI data analysis was carried out using fMRI Software Library (FSL) tools (Smith et al., 2004) as detailed below.

#### Task-based fMRI

Data preprocessing was carried out using fMRI Expert Analysis Tool (FEAT; Version 6.00; Woolrich et al., 2001). Preprocessing consisted of head motion correction, brain extraction, spatial smoothing using a Gaussian kernel of full width at half maximum (FWHM) of 5 mm, and high-pass temporal filtering equivalent to 90 s. fMRI volumes were registered to the individual’s structural scan and Montreal Neurological Institute (MNI) standard space images using both linear (FLIRT) and nonlinear (FNIRT) registration tools, then optimized using boundary-based-registration approach (Greve & Fischl, 2009). These transformations into the MNI template were applied to images of contrasts of interest and their variances. Preprocessing results of each of the study participants were visually inspected by a trained neuroscientist (NF) to ensure registration accuracy.

Task-based fMRI data analysis, for both auditory and visual stimulus presentation, was performed using FEAT (Version 6.00; Woolrich et al., 2001). Time series statistical analysis was carried out with local autocorrelation correction. A boxcar convolved with a gamma hemodynamic response function, and its temporal derivative was applied to each of the four explanatory variables: NC, NS, SC and SS. The main contrasts of interests were: (1) NC versus NS, (2) NC versus SC, (3) NC versus SS. Estimates for each of the four conditions versus implicit baseline were also obtained for reference. Higher level (group level) analysis was carried out using FMRIB’s Local Analysis of Mixed Effects (FLAME; Woolrich et al. (2004). Two separate general linear models (GLM) were used for the participants who underwent auditory or visual stimulus presentation. Here, we tested for group averages for each of the explanatory variables. Moreover, a separate GLM was used to compare the auditory versus visual groups for each of the contrasts of interest. Age was added as a nuisance variable (covariate of no interest), in order to account for potential nontask related effects that could influence our results.

#### Resting fMRI

Preprocessing was carried out using FEAT (Woolrich et al., 2001) in a similar fashion as performed for the task-based fMRI data. High-pass temporal filtering equivalent to 100 s was applied to resting fMRI data. As resting fMRI data are known to contain spurious signals, functional images were denoised to increase the possibility of identifying markers of effective connectivity. FMRIB’s ICA-based X-noiseifier (FIX) was applied (Griffanti et al., 2014) and a training dataset was specifically developed on the MOUS participants. The optimal threshold for the use of FIX, to denoise functional images, was identified as 20 (proportion of good vs. bad components) with a mean (median) true positive rate (TPR) and true negative rate (TNR) of 98.1% (100%) and 89.9% (90.2%), respectively. This is in line or above the suggested thresholding cutoffs (TPR > 95% − TNR > 70%). Preprocessed and denoised functional data for each subject were temporally concatenated across all subjects in order to create a single 4D dataset and to derive the population-based resting state networks (RSNs) using Multivariate Exploratory Linear Optimized Decomposition into Independent Components (MELODIC; Beckmann & Smith, 2004). The number of components was fixed to 25 based on an initial analysis of the population using model order estimation, which suggested that only 25 components were significantly non-zero on average. The between-subject analysis of the resting data was carried out using the dual regression approach, which allows for voxel-wise comparisons of resting functional connectivity maps (Beckmann et al., 2009; Filippini et al., 2009).

Analysis on resting fMRI data was performed in order to exclude that the auditory and visual groups had any underlying group disparities in functional connectivity potentially reflecting group-related differences in the task-based fMRI data.

#### Structural MRI

Preprocessing for structural images included the following steps: (a) re-orientating images to the MNI standard space template, (b) bias field correction, (c) brain extraction, and (d) brain tissues segmentation using FMRIB’s Automated Segmentation Tool (FAST) that allows generating maps and derive measures of total gray matter (GM), white matter, and cerebrospinal fluid for each individual subject.

In order to investigate potential differences in GM areas between the auditory group and the visual group, which could influence group-related differences in the task-based fMRI comparison, whole brain analysis was carried out using a voxel-based morphometry-style analysis (FSL-VBM; Douaud et al., 2007). Brain extraction and tissue-type segmentation were performed, and resulting GM partial volume images were aligned to the MNI standard space template using FLIRT and then FNIRT registration tools. A study-specific GM template was created. Images were averaged, modulated, and smoothed with an isotropic Gaussian kernel of 5 mm FWHM and the GM images were reregistered to the study-specific GM template, including modulation by the warp field Jacobian.

For data exploration we performed region-of-interest (ROI) analyses calculating percent signal change for the four simple contrasts (SW, CW, SS, CS). For ROI analyses we used ROIs based on a meta-analysis by Zaccarella, Schell, and Friederici (2017).

## RESULTS

### Study Participants

We identified 187 participants out of 204 that met the inclusion criteria. Ninety-two participants underwent auditory stimulation and 95 visual stimulation. A total of 17 subjects were discarded because of the following reasons: (1) the encoding direction for the task fMRI and/or for the resting fMRI acquisitions was different from the original setup (*N* = 12), (2) resting fMRI data missing (*N* = 2), and (3) problems with the log files (*N* = 3).

Participant characteristics for the respective groups were: auditory group (*N* = 92, age = 22.02[±3], males/females = 44/48) and visual group (*N* = 95, age = 21.89[±2.84], males/females = 44/51). No difference was observed between the two study groups for age and sex distribution. *T* tests and Chi-squared statistical tests were used to compare, respectively, age and sex distribution between the two study groups. No differences were observed, with *p* = 0.77 for age and *p* = 0.88 for sex distribution. Data analysis was carried out using SPSS statistics software (Version 28; IBM, n.d.).

### Imaging Results

#### Task-based fMRI

Task-based fMRI analyses, for the three GLMs (group average auditory and visual tasks, and group-related comparison for the auditory vs. visual task) revealed specific brain regions activated during the task. The *Z* statistic images were thresholded using clusters determined by *Z* > 3.1, and a family-wise error (FWE) cluster corrected significance threshold of *p* < 0.05 was applied to the suprathreshold clusters.

##### Comparison between contrasts per modality.

**Figure 3.**
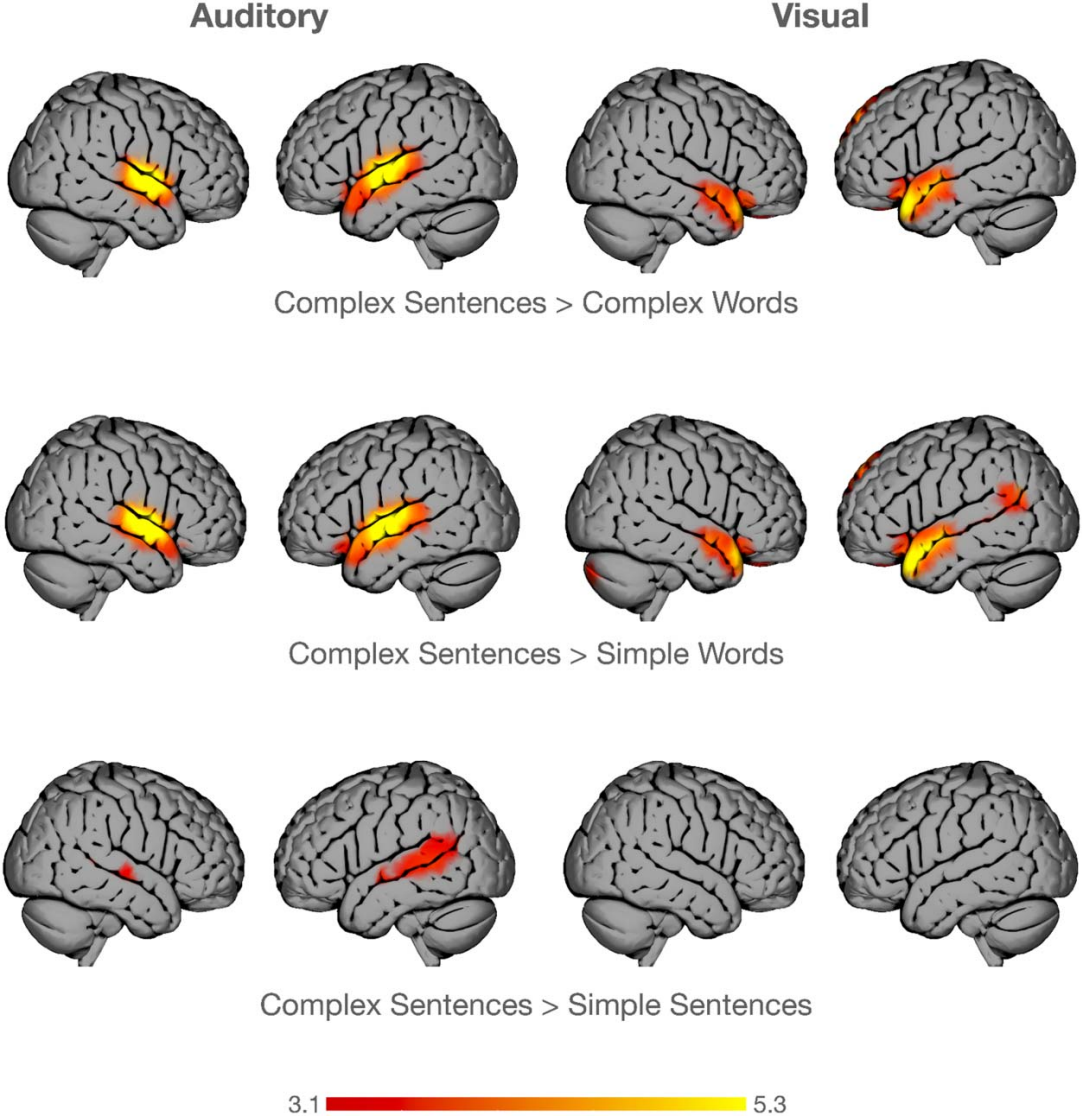
Comparison between contrasts per modality. Group-level fMRI statistical maps of the three main contrasts of interest, for auditory stimulation modality (left panel: *n* = 92) and the visual simulation modality (right panel: *n* = 95). Auditory stimulation led to BOLD increase for complex sentences (CS) in bilateral primary auditory cortices and medial superior temporal gyrus (STG) contrasting against complex words (CW) or simple words (SW), whereas it led to increased BOLD signal largely confined to the left posterior STG when contrasting against simple sentences (SS). In the visual condition the same contrasts led to an increase in bilateral anterior temporal lobes for CS against CW or SW and no BOLD increase above the statistical threshold for the CS vs. SS contrast. BOLD increases (*z*-values) are displayed between 3.1 and 5.3. Results are overlaid on the cortical surface. Axial slices for reference are displayed in the Supporting Information (Figure S1 and Figure S2).

Results are summarized in Figure 3. For the auditory stimulation, while contrasting complex sentences (CS) against complex words (CW) or simple words (SW) both resulted in bilaterally increased BOLD activity in the primary auditory cortices and medial STG, contrasting complex (CS) against simple sentences (SS) revealed stronger activation changes in the posterior left STG. Regarding the visual stimulation, contrasting CS against CW or SW both resulted in bilateral activation increase in the temporal poles and additionally in increases in the posterior part of the STG. Contrasting CS against SS revealed no significant change in BOLD response. Statistics for main clusters are reported in Table 1. For a combined model over modalities see Figure S3 in the Supporting Information, available at https://doi.org/10.1162/NOL.a.25.

**Table 1.** Main clusters for comparisons between contrasts per modality

Contrasts	Cluster size	*Z*-max	MNI peak coordinates in voxel
Auditory			
CS > CW	3,341	10.6	−58, −12, 4
CS > SW	3,821	10.7	−58, −12, 4
CS > SS	1,360	4.99	−50, −38, 2
Visual			
CS > CW	3,202	7.1	−54, −6, −10
CS > SW	4,032	7.3	−46, 18, −28
CS > SS	ns	ns	ns

*Note*. CS = complex sentence, CW = complex word, SW = simple word, SS = simple sentence, ns = nonsignificant, MNI = Montreal Neurological Institute.

##### Contrasts between modalities.

**Figure 4.**
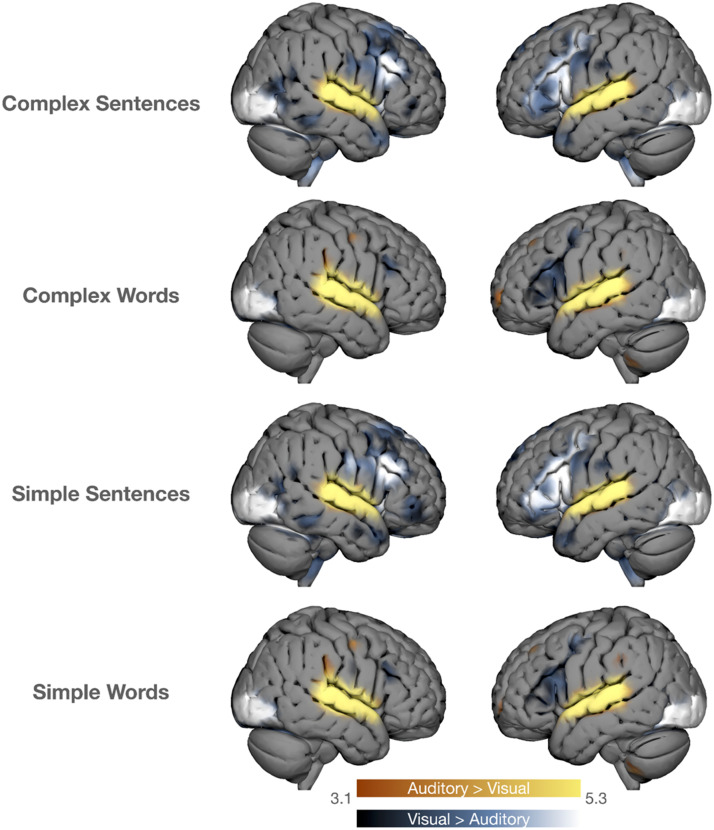
Contrasts between modalities for each condition. Group-level fMRI statistical maps contrasting the auditory stimulus modality against the visual stimulus modality and vice versa for each condition (CS, CW, SS, and SW). While the auditory vs. visual contrast revealed only BOLD increase in primary auditory areas (A1), the opposite contrasts resulted in higher inferior frontal gyrus (IFG) activation additionally to primary visual processing (V1). BOLD increases (*z*-values) are displayed between 3.1 and 5.3. Results are overlaid on the cortical surface.

Contrasts are depicted in Figure 4. Contrasting auditory > visual stimulation for all four conditions (i.e., CS, CW, SS, and SW) revealed a similar activation pattern in the bilateral STG. Concerning the opposite contrast visual > auditory there was a significant BOLD increase in the primary visual cortex for all four conditions. However, CS and SS additionally resulted in bilateral activation increase in the IFG and posterior portion of the ventral temporal lobe, whereas CW and SW resulted in less pronounced and stronger lateralized activation changes in the IFG.

##### Contrasts of interest against baseline.

Contrasts are depicted in Figure S4. Contrasting Auditory stimulation against implicit baseline for all four conditions (CS, CW, SS, and SW) revealed a similar activation pattern in the bilateral STG. Concerning the Visual against implicit baseline contrast, there was a significant BOLD increase in the primary visual cortex and posterior STG for all four conditions. However, CS and SS additionally resulted in bilateral activation increase in the left primary motor cortex.

#### Resting fMRI

Here, we investigated functional connectivity differences between our two study groups, potentially underlying group-related (auditory vs. visual modality of stimuli delivery) effects on fMRI task-based data. Among the 25 derived RSNs, we selected for our analyses four RSNs of interest, namely, the medial visual (MV) and lateral visual (LV) networks, the auditory (AU) network, and the language (LA) network. The rationale for this was the following: (1) the MV and LV RSNs include occipital brain regions in the visual cortex that are activated during the visual modality of stimulus delivery; (2) the AU RSN includes primary and secondary auditory cortices involved during the auditory modality of stimuli delivery; and (3) the LA network includes brain regions involved in language processing that are activated as part of the specific task used here.

Voxel-wise GLM was applied on RSN maps using a permutation-based nonparametric testing, Randomise (*N* = 2,000 permutations; Winkler et al., 2014), and Threshold Free Cluster Enhancing (TFCE) for clusters identification (Smith & Nichols, 2009). FWE corrected cluster significance threshold of *p* < 0.05 was applied to the suprathreshold clusters. No RSN differences were observed between the two study groups suggesting that task-based fMRI group-related effects reported here reflected stimulus delivery, and they were not dependent on any underlying functional connectivity difference between the auditory and the visual group.

#### Structural MRI

Group voxel-wise GLM was applied using Randomise (*N* = 2,000 permutations; Winkler et al., 2014) and threshold-free cluster enhancement (Smith & Nichols, 2009). Then an FWE corrected cluster significance threshold of *p* < 0.05 was applied to the suprathreshold clusters. No GM differences were observed between the two study groups.

#### ROI analysis

Boxplots for percent signal change in the selected ROIs revealed that the majority of conditions did not result in an activation increase (signal change below or around zero) and variability was high between participants. Results plots are found in the Supporting Information (Figure S1 and Figure S2).

## DISCUSSION

In this study, we have shown the impact of control condition (semantic, syntactic) and stimulation modality (visual, auditory) leveraging a large repository dataset (MOUS; Schoffelen et al., 2019). The exemplary dataset focused on syntactic processing. Here, we could show that (1) there is a large difference in outcome measures for auditory as opposed to visual stimulation, (2) control conditions strongly impact outcomes, and (3) there is an additional influence regarding control condition by stimulation modality.

### Stimulation Modality

Concerning auditory stimulation, our results are in line with a recent meta-analysis (Turker et al., 2023), where the middle bilateral portion of the STG was shown to be strongly involved in all language tasks. Interestingly, this effect is very prominent considering *z*-values higher than 10 in our analyses. While for the auditory condition comparison between complex and simple sentences led to an activation increase in the posterior superior temporal sulcus (STS), the other two contrasts (comparing CS to CW or SW) resulted in bilateral middle STG activation increases. This hints toward the fact that comparison with auditory word strings might not result in more activation changes as explained by primary auditory stimulation, while the comparison to structured but simpler sentences revealed activation changes in line with higher complexity assumptions.

For the visual condition, we found a more divergent pattern across contrasts: (a) a comparison of CS to both scrambled-words conditions, SW and CW, led to bilateral increase of temporal pole activation, and (b) there was no significant difference between CS and SS. The temporal pole has been associated with story reading and especially with semantic processing (Borghesani et al., 2016; Herlin et al., 2021). Regarding direct comparison between modalities, unsurprisingly, the primary visual cortex was more strongly involved during visual relative to auditory presentation. Beyond these patterns, it turned out that there was a stronger involvement of the bilateral IFG during sentence processing for the visual compared to the auditory modality. Scrambled words (SW, CW), on the other hand, resulted in a less pronounced but left-lateralized activation change in the IFG. While the involvement of the IFG highlights the processing of syntactic structures (Turker et al., 2023; Tyler et al., 2009; Zaccarella, Meyer, et al., 2017; Zaccarella, Schell, & Friederici, 2017), the larger involvement of right hemispheric IFG areas might suggest a higher cognitive demand (Hartwigsen et al., 2021). It can also be argued that stronger activation in the left IFG during the visual modality compared the the auditory modality is a result of its role in motor planning related to language. More specifically, the left IFG has been associated with pronunciation (Golestani et al., 2011). The increased engagement of the left IFG may therefore reflect higher levels of implicit articulation during reading. Hence, a stronger involvement of the IFG during reading might have been the result of the more productive nature of the reading task as compared to attentive listening (Indefrey et al., 2004; Turker et al., 2025).

Taken together, auditory stimulation seems to have a pronounced effect on BOLD response that—at least in the MOUS dataset—was so prominent that it masked the influence of other language related functions. On the other hand, visual stimulation allowed for more insightful effects. However, a strong involvement in the expected IFG was shown only by contrasting the two modalities against each other. This is surprising given that the IFG has been repeatedly found activated during structural contrasts in previous studies (Zaccarella, Schell, & Friederici, 2017).

### Control Conditions

In general, using word stimuli, irrespective of whether they were created from complex or simple sentences, did not result in a considerable change in contrasts of interests. However, correcting for semantic processing, by scrambling sentences to word lists, or syntactic complexity, by omitting a relative clause, resulted in strong variations in activation patterns. An unexpected result is the fact that contrasting complex sentences against simple and complex words resulted in an increase in the temporal pole for visual processing, which might indicate stronger semantic processing, but did not seem to fully subtract the effect of nonsyntactic processing. Artificially scrambled stimuli might provoke attentional shifts, leading to brain responses that are not indicative of those caused by natural stimuli (Chen et al., 2023). Thus, scrambled words might have triggered the urge to process a structure in the stimulus potentially resulting in similar brain responses as opposed to grammatical sentences. The exposure to syntactically easier sentences did not result in any significant difference in the visual but in the auditory condition, though hinting more toward an increase in phonemic processing (posterior STG). These results suggest that there is in general a weak effect of complex syntactic processing for purely paying attention to the stimuli either during visual or auditory stimulation.

### Comparison With MOUS MEG Study Results

In the original MOUS study, the same participants additionally performed the same task during magnetoencephalography (MEG) for high temporal resolution electrophysiological measurements. Exploring these MEG data, Arana et al. (2020) found an overlap across modalities in the temporal window, within which supramodal cortical areas are activated. In contrast to our study, in which auditory processing solely activated the bilateral STG, Arana et al. (2020) found that supramodal activity included parts of the left temporal cortex, left inferior parietal lobule, as well as the prefrontal cortex, becoming apparent as early as 250 ms and lasting until 700 ms after word onset. This suggests that prominent bilateral activation in the STG during auditory stimulation could indeed have masked the influence of other language related functions in the fMRI dataset.

Comparing our findings to those of Arana et al. (2020), it becomes apparent that MEG found mostly left-lateralized involvement of both the STG and the IFG. In contrast, fMRI picked up on bilateral STG during the auditory modality as well as bilateral IFG involvement during the visual modality.

### The Role of IFG in the Context of Syntactic Processing

The role of the IFG in the context of structural processing is of strong theoretical interest (Friederici, 2017; Hagoort, 2013). In contrast to established research (Zaccarella, Schell, & Friederici, 2017), the present dataset did not reveal any significant activation change in the IFG by looking into results separately for modalities. Modality and task demand specificity of the IFG became apparent only by directly comparing the two modalities against each other, resulting in expected increases in IFG activations for sentence structures during the visual modality in line with previous literature (Rogalsky et al., 2015; Rogalsky & Hickok, 2011). In this context it has been argued that IFG activations during syntactic processing might originate from the modality itself (Indefrey et al., 2004). On the other hand, Fedorenko et al. (2010) showed similar activation patterns for both modalities, including the frontal cortex, in a similar study. In terms of results on syntactic processing in the IFG the present sample generally differs from previous findings.

### Summary

Leveraging the MOUS dataset in order to systematically vary the influence of control condition and stimulation modality strongly suggests that using auditory stimulation predominantly results in BOLD increase in primary auditory cortices. This result might also reflect the overall difficult sound environment in the MR scanner (Pellegrino, Schuler, et al., 2022). Further research should investigate the extent to which primary sensory stimulation during fMRI masks task-related effects of interest.

## ACKNOWLEDGMENTS

The contribution from NF to this work is supported by the Italian Ministry of Health (Ricerca Corrente). The authors are grateful to the scientists involved in the collection of the Mother of Unification Studies (MOUS) dataset and for making it available online.

## FUNDING INFORMATION

Nicola Filippini, Ministero della Salute (Ricerca Corrente) (https://dx.doi.org/10.13039/501100003196).

## AUTHOR CONTRIBUTIONS

**Anna-Lisa Schuler**: Conceptualization; Formal analysis; Visualization; Writing – original draft; Writing – review & editing. **Ella Teuscher**: Visualization; Writing – original draft; Writing – review & editing. **Nicola Flippini**: Conceptualization; Formal analysis; Funding acquisition; Methodology; Project administration; Resources; Writing – original draft; Writing – review & editing.

## DATA AND CODE AVAILABILITY

All data is publicly available at https://data.donders.ru.nl. No built in-house code or AI tools were used for the analysis of the fMRI data presented in our manuscript. All commands adopted here were part of the publicly available FSL package and they were fully described in the Materials and Methods and Results sections, including the specific options employed for each command.

## Supplementary Material



## TECHNICAL TERMS

Functional magnetic resonance imaging (fMRI): Method that uses strong magnetic fields to identify brain activity based on oxygen content of blood.
Inferior frontal gyrus (IFG): Brain area in the frontal cortex that is involved in language production.
Magnetoencephalography: Method that uses magnetic fields to measure electromagnetic cortical activity.
BOLD signal: Signal based on blood oxygenation level; signal for fMRI.
Generalized linear model (GLM): A statistical model based on linear contrast estimates.
