# Supplementary material for: Implications of Stimulation Modality and Control Condition on BOLD Response: An Example From the MOUS Dataset
Source: Neurobiol Lang (Camb). 2025 Dec 1;6:NOL.a.25. doi: 10.1162/NOL.a.25 (PMC12674533; doi:10.1162/NOL.a.25)
Supplement: Supplementary file 1 [file nol-6-1-25-s001.pdf]

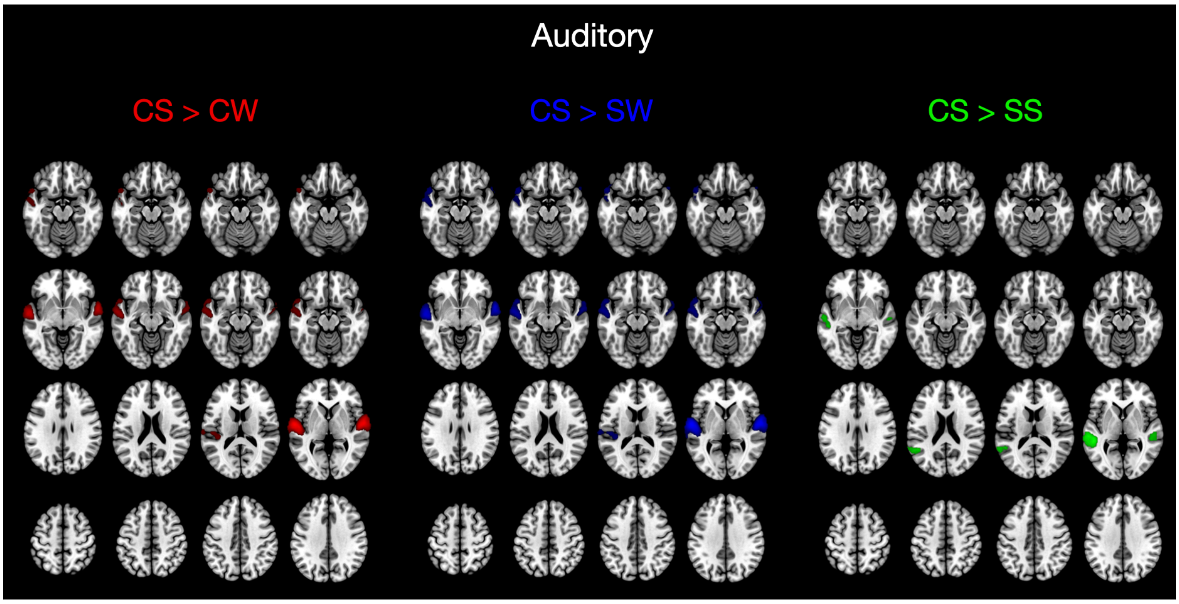

Figure S1. Axial view of the three contrasts for the auditory modality. Overlays are displayed from zero.

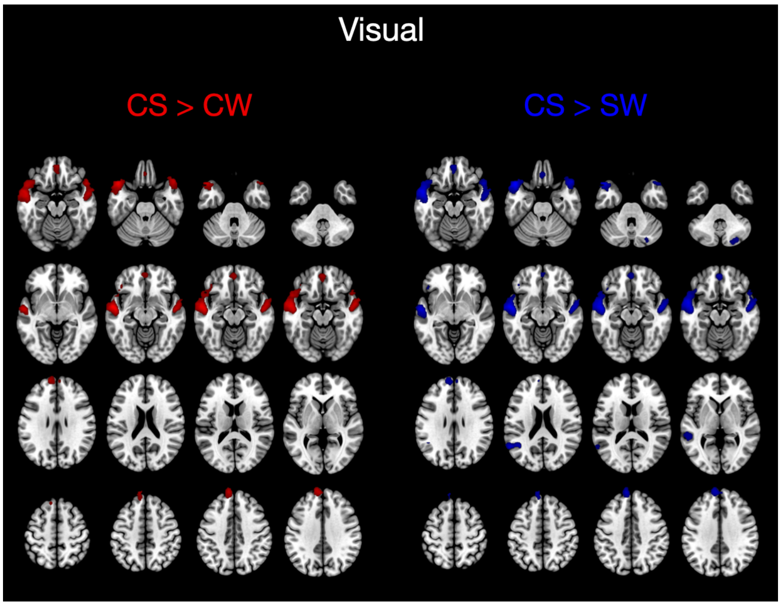

Figure S2. Axial view of the three contrasts for the visual modality. Overlays are displayed from zero.

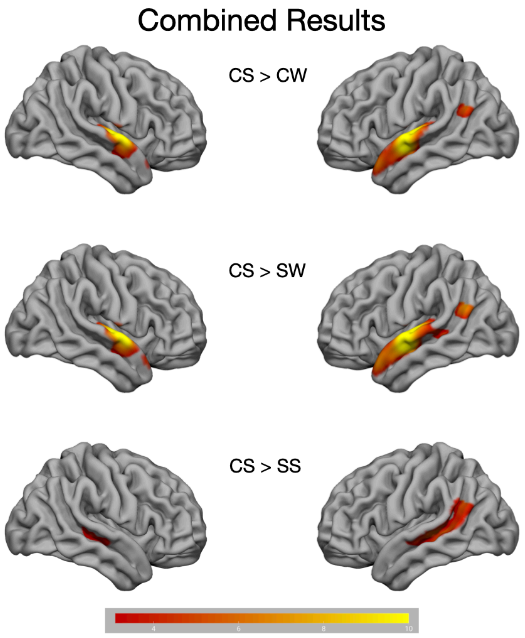

Figure S3. Three main contrasts of interest over both modalities. CS>CW and CS>SW contrasts mainly showed activation increases for bilateral middle part of superior temporal gyrus with an additional cluster in the left angular gyrus. CS>SS contrast revealed bilateral activation increase in the middle to posterior part of the superior temporal gyrus. Color map represents t-values (3.1 to 10).

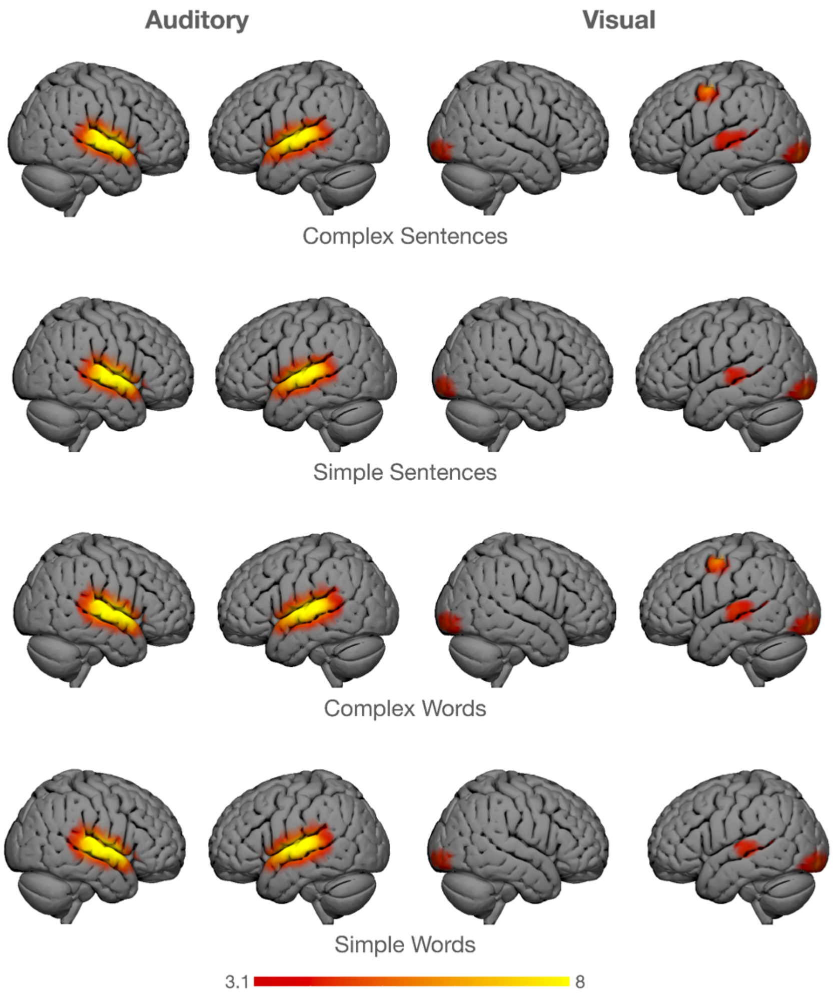

Figure S4. Conditions against Baseline for each Modality. fMRI statistical maps of the four conditions contrasted to brain activity without stimulation (baseline) for auditory stimulation modality (left panel: n = 92) and visual simulation modality (right panel: n = 95). Auditory stimulation resulted in significant increases in primary auditory cortices, while visual stimulation led to BOLD increase in the more posterior part of the left superior temporal gyrus (STG). BOLD increases (z-values) are displayed between 3.1 and 8.0. Results are overlaid on the cortical surface.

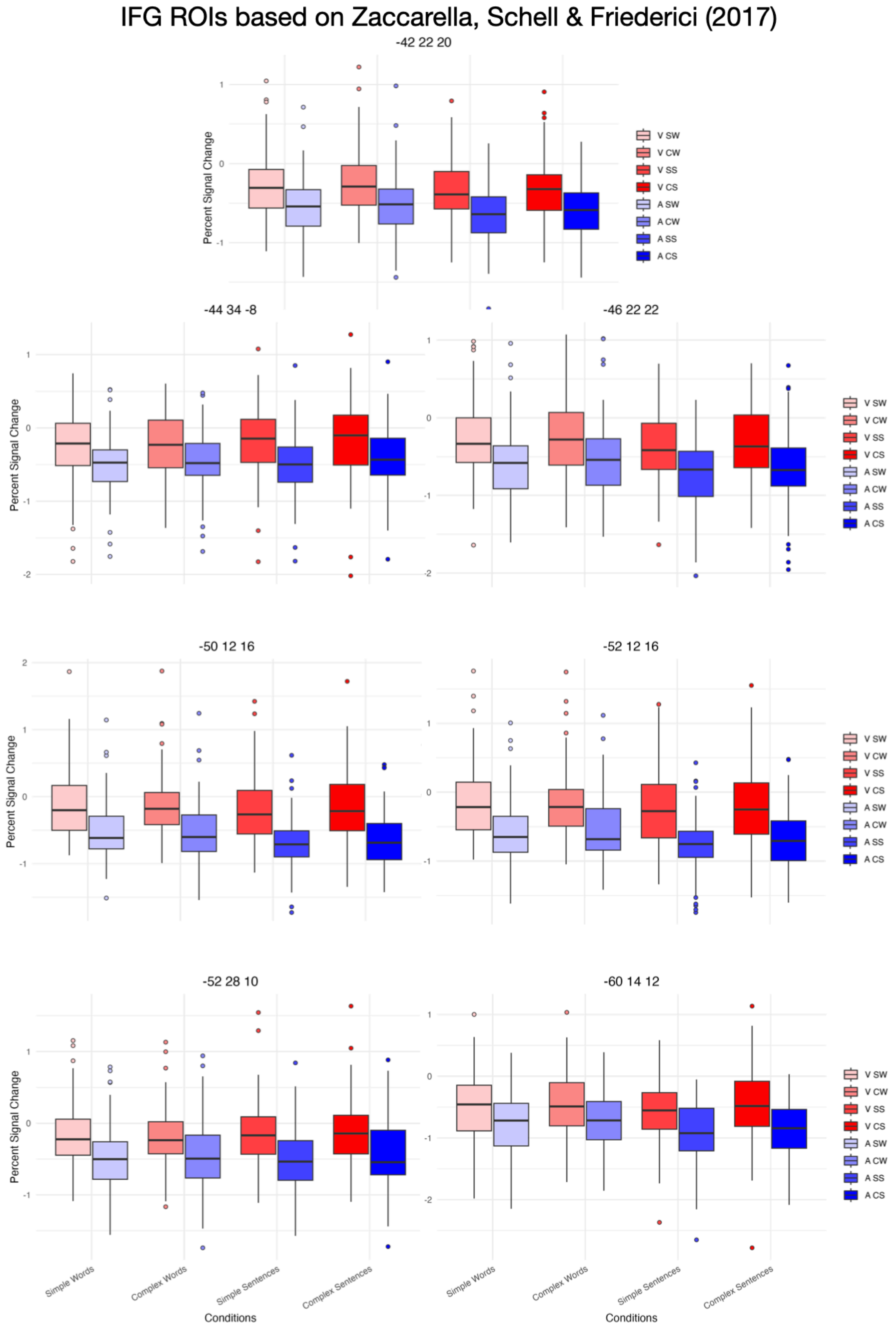

Figure S5. Frontal ROI results based on Zaccarella, Schell & Friederici (2017). ROIs were based on the "Licit structures vs. word-list controls" and "Content OR Function word-list studies" ALEs of the referenced publication. Percent signal change was below zero in all ROIs and conditions. Red gradients describe the visual condition while blue colors describe the auditory conditions.

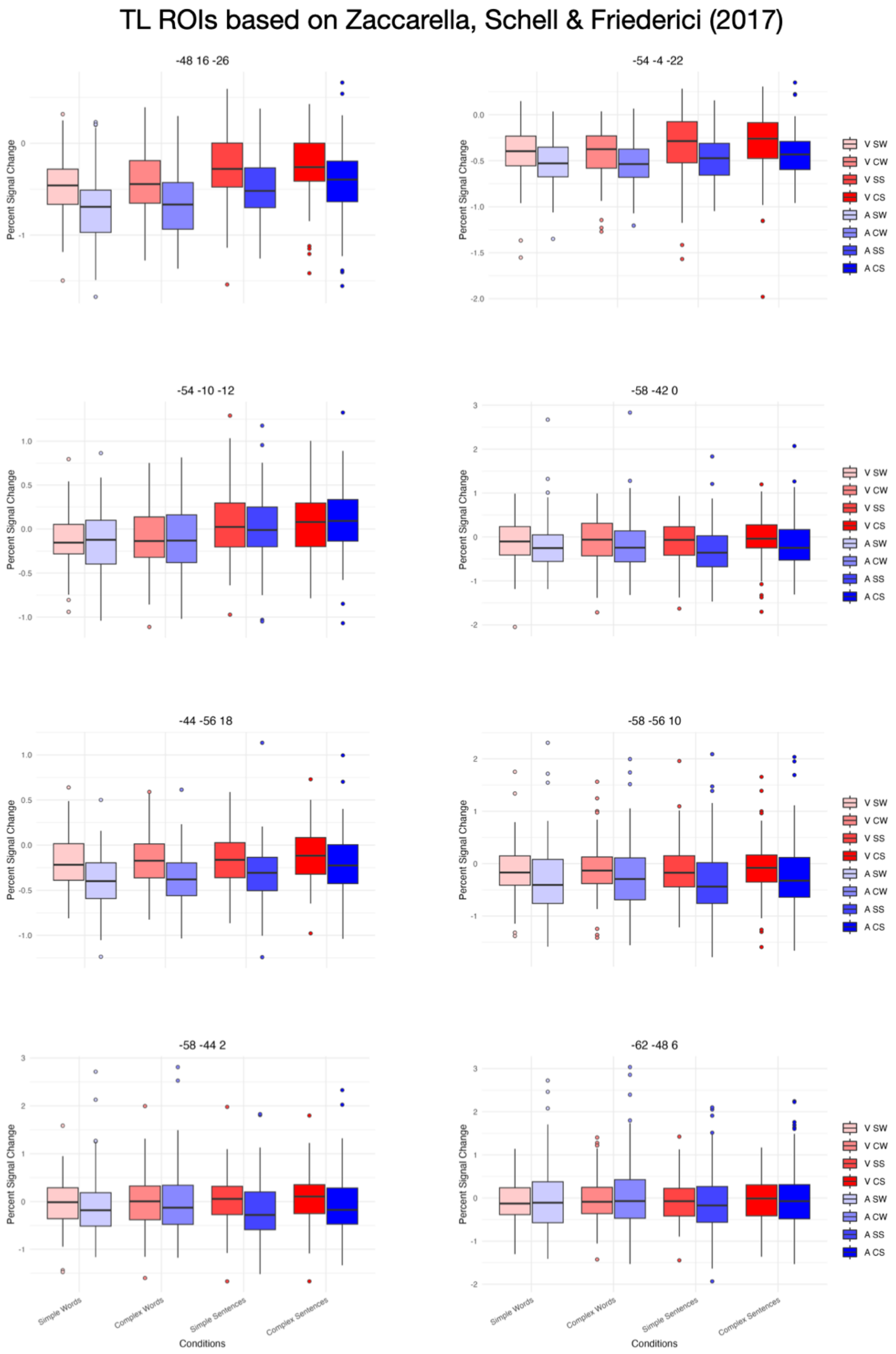

Figure S6. Temporal ROI results based on Zaccarella, Schell & Friederici (2017). ROIs were based on the “Licit structures vs. word-list controls” and “Content OR Function word-list studies” ALEs of the referenced publication. Percent signal change was below zero or close to zero in all ROIs and conditions. Red gradients describe the visual condition while blue colors describe the auditory conditions.

References

Zaccarella, E., Schell, M., & Friederici, A. D. (2017). Reviewing the functional basis of the syntactic Merge mechanism for language: A coordinate-based activation likelihood estimation meta-analysis. *Neuroscience & Biobehavioral Reviews*, *80*, 646-656.
